# Inflammatory microenvironment in classical Hodgkin’s lymphoma with special stress on mast cells

**DOI:** 10.3389/fonc.2022.964573

**Published:** 2022-10-13

**Authors:** Domenico Ribatti, Roberto Tamma, Tiziana Annese, Giuseppe Ingravallo, Giorgina Specchia

**Affiliations:** ^1^ Department of Basic Medical Sciences, Neurosciences and Sensory Organs, University of Bari Medical School, Bari, Italy; ^2^ Department of Medicine and Surgery, Libera Università del Mediterraneo (LUM) Giuseppe Degennaro University, Bari, Italy; ^3^ Department of Emergency and Transplantation, Pathology Section, University of Bari Medical School, Bari, Italy; ^4^ Department of Emergency and Transplantation, Hematology Section, University of Bari Medical School, Bari, Italy

**Keywords:** classical hodgkin’s lymphoma, mast cells, tumor microenvironment, angiogenesis, inflammatory cells

## Abstract

Classical Hodgkin’s lymphoma (CHL) accounts for 10% of all lymphomas. Nodular sclerosis and mixed cellularity accounts for nearly 80% of all CHL cases. The number of mast cells in CHL correlates with poor prognosis, is significantly higher in nodular sclerosis than in other CHL subtypes, and an association between the degree of angiogenesis and the number of intratumoral mast cells has been demonstrated in CHL. Even with the best available treatment, a significant percentage of CHL patients progress or relapse after first-line therapy. 50% of patients with disease relapse achieve subsequent long-term disease control with salvage therapies. In this context, new potential therapeutic opportunities are required, and mast cells may be regarded as a new target for adjuvant treatment of CHL through the inhibition of angiogenesis and tissue remodeling and allowing the secretion of cytotoxic cytokines.

## Introduction

Classical Hodgkin’s lymphoma (CHL) accounts for 10% of all lymphomas. Four subtypes of HL are recognized: nodular sclerosis (the most common histologic form), mixed cellularity (more patients with this form present disseminated disease), lymphocyte predominance, lymphocyte depletion (very rare). Nodular sclerosis and mixed cellularity accounts for nearly 80% of all CHL cases (1). Nodular sclerosis subtype is characterized by the presence of extracellular matrix deposits consisting of collagen-rich fibrotic bands surrounding aggregates of inflammatory and neoplastic cells.

The characteristic neoplastic cell of CHL is the Reed-Sternberg cell (RSC), a large cell with two or more nuclei or nuclear lobes each of which contains large eosinophilic nucleolus. RSCs are common in the mixed cellularity subtype, uncommon in the nodular sclerosis subtype, and rare in the lymphocyte predominance subtype. RSCs and Hodgkin’s cells (HCs) corresponds to just 1% to 10% of the total tumor mass; the remaining consists of T and B cells, plasma cells, neutrophils, eosinophils, macrophages, mast cells, myeloid derived suppressor cells (MDSCs), fibroblasts and endothelial cells (1).

RSCs synthesize and release interleukin (IL)-5, -7, -8, -9, -13, chemokine (C-C motif) ligand (CCL)-5, -17, -20 and -22 that are involved in the recruitment of granulocytes, lymphocytes, mast cells, and macrophages (2). Transforming growth factor beta (TGFβ) produced by RSCs plays an important role in CHL-associated fibrosis, as demonstrated by immunohistochemistry and *in situ* hybridization (3, 4). IL-13 produced by RSCs stimulates the synthesis of TGFβ by macrophages (5) or may directly initiates fibrosis as well as stimulates mast cell proliferation and infiltration (6).

Vascular endothelial growth factor (VEGF), matrix metalloproteinases-2 and -9 (MMP-2 and MMP-9), and tissue inhibitor of MP-1 (TIMP-1) are expressed in RSCs in childhood HL, but microvascular density was not correlated with the expression of these factors (7). A retrospective study on CHL, demonstrated that VEGF-A, VEGF receptor-1 and -2 (VEGFR-1 and VEGFR-2) are expressed by RSCs and lymphocytes (8).

In this review article, we will summarize the literature concerning the role of inflammatory cells in tumor microenvironment in CHL with particular focus on mast cells and will suggest a potential use of inhibitors of mast cells as adjuvant therapeutic approach in the treatment of CHL.

## Inflammatory cells in tumor microenvironment in CHL

**Figure 1:**
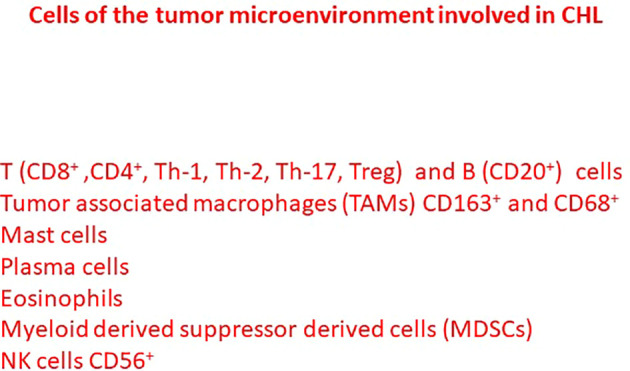
Different cells of the tumor microenvironment involved in the pathogenesis and tumor progression of classical Hodgkin lymphoma.

The CHL tumor microenvironment includes a rich inflammatory cellular component, including T and B cells, tumor associated macrophages (TAMs), mast cells, plasma cells, eosinophils, MDSCs and NK cells, secreting cytokines and chemokines involved in the regulation of tumor initiation as well as its progression and metastasis. Scientific works highlight the importance of the tumor microenvironment composition in determining the pathogenesis of CHL and the complex interplay between the different cells of tumor microenvironment (**Figure 1**).

Several T cell subsets [Th-1, Th-2, Th-17, T regulatory (Treg) cells and cytotoxic lymphocytes] are distributed in the CHL tumor microenvironment. CD8^+^ and CD4^+^ T cells are the most represented component of CHL microenvironment. TH-2 and IL-10 secreting Treg cells are involved in the development of an immunosuppressive microenvironment (9). CD8^+^ and CD4^+^ T cells are more numerous around and in contact with PDL-1^+^ TAM than PD-L1^-^ TAMs suggesting that the PD-L1^+^ TAMs may either promote anti-tumor immunity through antigen presentation or immunosuppression through the engagement of PD-1 (10).

Non-malignant B cells are prevalent in nodular lymphocyte predominant CHL, which can be successfully treated with anti-CD20 monoclonal antibodies (11). High CD20^+^ cells predicted a favorable outcome in CHL and depletion of CD20^+^ cells together with an increase of TAMs identifies a group of patients with high-risk disease (12). CHL patients with high proportion of plasma cells have an inferior event free survival and overall survival although significance was not maintained in the multivariate analysis (13).

**Figure 2:**
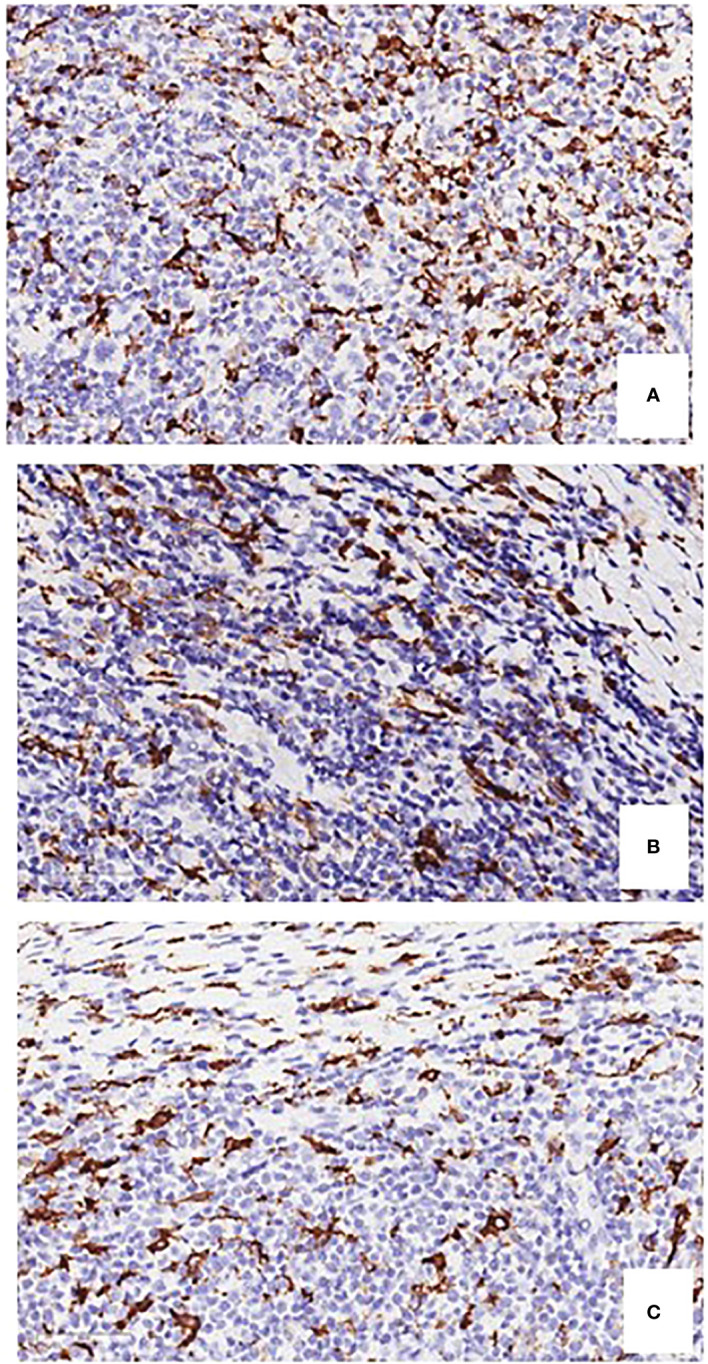
Immunostaining of CD163-positive and CD68-positive macrophages **(A, B)**, and tryptase-positive mast cells **(C)**, in bioptic specimens of human classical Hodgkin’s lymphoma. Original magnification, 400 x.

CD163 expression in TAMs is correlated with angiogenesis and shortened survival in CHL, suggesting an interaction between RSCs and TAMs (**Figure 2A**) (14). PD-L1^+^TAMs and PD-1^+^ CD4 T cells are in contact with PD-L1^+^ tumor cells (10). A higher number of CD68^+^ TAMs is associated with shortened survival and with the outcome of secondary treatments (**Figure 2B**) (15, 16). PD-L1^+^ TAMs and PD-L1^+^ CD4^+^ T cells are significantly more abundant in the proximity of RSCs (10). RSCs and HCs recruit monocytes from the blood and can induce by the secretion of IL-10, -IL-13 CCR-5, tumor necrosis factor (TNF), and granulocyte macrophage colony stimulating factor (GM-CSF), a TAM M2 phenotype, which suppress cytotoxic T cells and recruit T-reg cells (16).

CHL contains low levels of CD56^+^ NK cells and few studies indicated a dysregulation of NK function (17). A high number of arginase 1-positive MDSCs is significantly associated to shorter progression free survival and to a worse overall survival (18). In CHL patients showing complete and partial responses in a phase I trial using the PI3Kγ/δ inhibitor RP6530, a significant inhibition of circulating MDSCs was demonstrated (19).

## Mast cell staining and ontogeny

For the first 60 years after Ehrlich’s discovery of mast cells in 1878, the study of these cells was almost entirely histological. Their single nucleus shows a round or oval shape and the cytoplasm contains numerous secretory granules that metachromatically stain with thiazine dyes such as toluidine blue. Metachromatic staining is important in the detection of mast cells and is strongly recommended as a routine stain. Mast cells highlighted with toluidine blue may be counted counted in 6-8 250 x fields, covering almost the whole section, inside a superimposed square reticulum (0.25 mm^2^), and calculated as means ± 1 standard deviation for each group of samples (20).

Mast cells have their origin in the bone marrow and develop along the myeloid pathway. Demonstration of mast cell derivation from bone marrow precursors could be established in 1977 when Kitamura’s group first showed reconstitution of mast cells in mast cell-deficient mice by the adaptive transfer of wild type bone marrow and indicated that these cells were of hematopoietic origin (21). Human mast cells originate from CD34 ^+^/CD117^+^/CD13^+^ multipotent hematopoietic progenitors in bone marrow that migrate through blood to tissues where they differentiate. However, many aspects of their differentiation and phenotypic diversification are still understood.

## Mast cells in CHL

For many years mast cells have been considered involved in several allergic disorders. More recently, it has been clearly established that mast cells and their mediators are involved in different aspects of tumor initiation and growth. Mast cells impact tumor cells as well as immune and non-immune components through chemokine secretion and release of other mediators, with cancer-promoting or cancer-suppressive properties.

**Figure 3:**
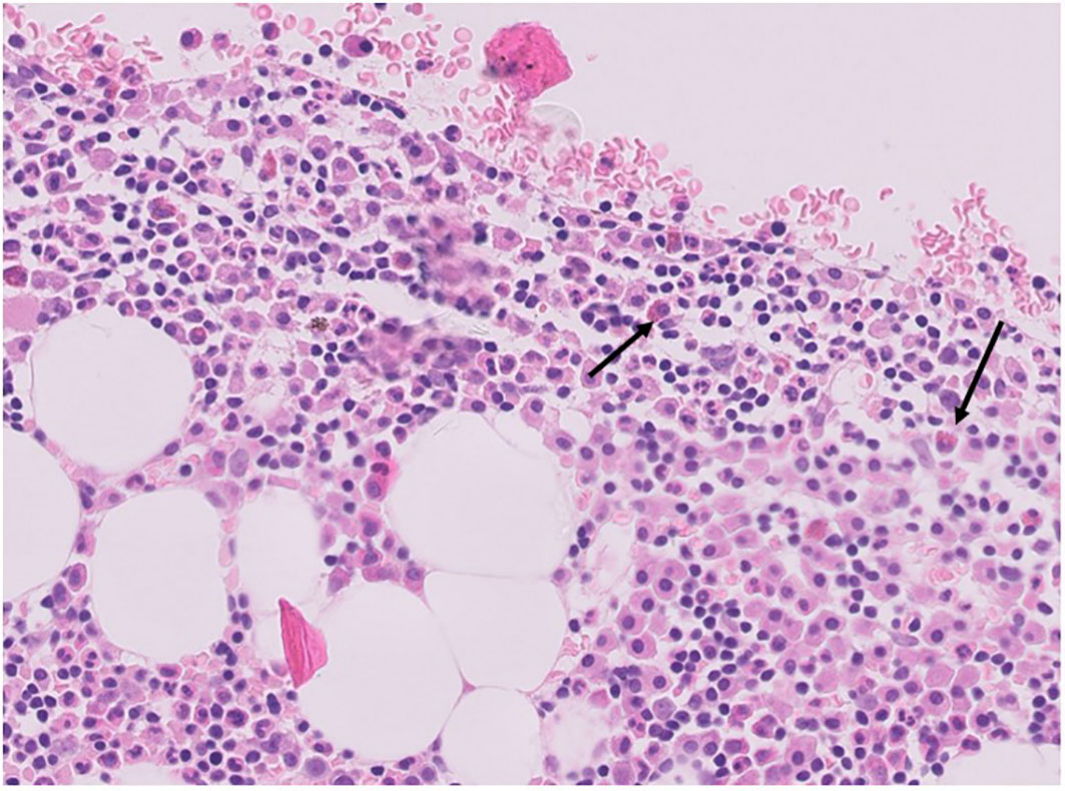
Hematoxylin & Eosin staining of mast cells (arrows) in a human CHL bioptic specimen (courtesy of professor Francesco Pezzella, Oxford).

Mast cells may promote tumor growth through the release of pro-angiogenic factors, the release of proteases able to affect stromal composition, and the release of tumor-promoting cytokines, including IL-10 and TGFβ (22). Mast cells can be visualized in CHL specimens with a conventional hematoxylin & eosin staining (**Figure 3**). The number of mast cells in CHL correlates with poor prognosis (23, 24), and is significantly higher in nodular sclerosis than in other CHL subtypes (**Figure 2C**) (25, 26). An association between the degree of angiogenesis and the number of intra-tumoral mast cells has been demonstrated in CHL (27).

Mast cells stimulate RSCs lines *in vitro* by CD30 ligand-CD30 interaction, suggesting that mast cells may promote tumorigenesis through direct interaction between CD30 antigen on RSCs and CD30 ligand on mast cells (24).

Mast cells are involved in the synthesis of TGFβ and IL-13, favoring fibrosis in CHL (6, 28). Mast cells were scattered in the fibrotic areas within the lymph node specimens from CHL patients stained with toluidine blue and a significantly positive correlation was observed between the rate of fibrosis and the number of mast cells (6). Tumors growing in immunodeficient NOD/SCID mice inoculated with both mast cells and RSCs are more fibrotic and vascularized as compared with mice inoculated with RSCs alone (29). TGFβ+ mast cells were significantly higher in nodular sclerosis CHL and the number of mast cells positively correlated with the rate of fibrosis (6). Fibrosis favors cancer progression and tumor cell invasion through the increase the local concentration of growth factors and cytokines, and in this context, fibrosis induced by mast cells could contribute to CHL progression. Finally, mast cells can promote the differentiation of T cells into FOXP3^+^/CD25^+^ Treg cells *via* the secretion of TGFβ and may be the major source of pro-tumorigenic angiogenic and lymphangiogenic factors (30).

## Therapeutic targeting of mast cells in CHL

The main treatments of CHL include multi-agent chemotherapy and involved field radiation therapy, which can significantly reduce the rate of death and the long-term remission of CHL can achieve 85%–95%. However, approximately 20%–30% of patients will be seen with disease progression or death within 5 years. The best-known drugs are anti-CD30 inhibitors and PD-1 antibodies, showing promising results for patients with relapsed and refractory CHL (31). Anti-CD30 inhibitors have been approved for the treatment of CHL patients after failure with autologous stem cell transplantation or multi-agent chemotherapy regimens (32), but only approximately 17% to 29% of those patients who experienced failure or relapsed with CD30-targeted therapy and autologous stem cell transplantation (ASCT) can expect complete remission (CR) after accepting the treatment of PD-1 antibodies (33).

Even with the best available treatment, a significant percentage (10% to 30%) of CHL patients progress or relapse after first-line therapy (34). 50% of patients with disease relapse achieve subsequent long-term disease control with salvage therapies (35). In this context, new potential therapeutic opportunities are required.

Treatment failure in CHL underscores the need of novel biomarkers and new therapeutic approaches to treat non-responding and relapsed patients. The cellular components of the tumor microenvironment may be an important target in the development of novel therapeutic strategies. Identification and targeting of mast cells represent an attractive therapeutic approach in cancer. New targeted anti-cancer therapies exert their effects on mast cells (**Table 1**). Mast cells can be therapeutically targeted by decreasing cell numbers through c-KIT inhibition, modulating mast cell activation and phenotype [through mast cell stabilizers, Fcε signaling pathway activators/inhibitors, antibodies targeting inhibitory receptors and ligands, tool like receptor (TLR) agonists], and altering secreted mast cell mediators and their downstream effects.

**Table 1:** Anti-tumor drugs that target mast cells.

Drug	Main target in mast cell
Imatinib mesylate (Gleevec, ST1571)	c-kit
Sorafenib	c-kit
Sunitinib	c-kit
Pazopanib (GW786034)	c-kit
Axitinib	c-kit
Dasatinib	c-kit
Enzastaurin	PKC-beta
Alemtzumab (Campath)	CD52
CpG activator (Promune)	TLR9
MDX 060	CD30 (ligand for CD301)
Tanespimycin (17AAG)	Heat shock 90 beta

In a retrospective study involving 37 patients with CHL, pre-treatment serum levels of VEGF, fibroblast growth factor-2 (FGF-2), hepatocyte growth factor (HGF) were measured and compared to post-therapy levels. Elevated pre-treatment VEGF and HGF levels were significantly reduced after therapy and both pre- and post-therapy VEGF levels were predictive of survival (36). Moreover, elevated VEGF pre-treatment levels were correlated with the tumor burden and continued to be elevated in prolonged complete remission.

Mast cells express high levels of c-KIT and its ligand stem cells factor (SCF). SCF enhances tumor growth through the production of VEGF, IL-6, IL-10, and TNFα (37). VEGF is a crucial molecule involved in angiogenesis in CHL (38). Bortezomib inhibits mast cell degranulation and the release of VEGF in CHL, and bortezomib-treated mast cells lose the ability to induce fibrosis and angiogenesis in CHL (29).

**Figure 4:**
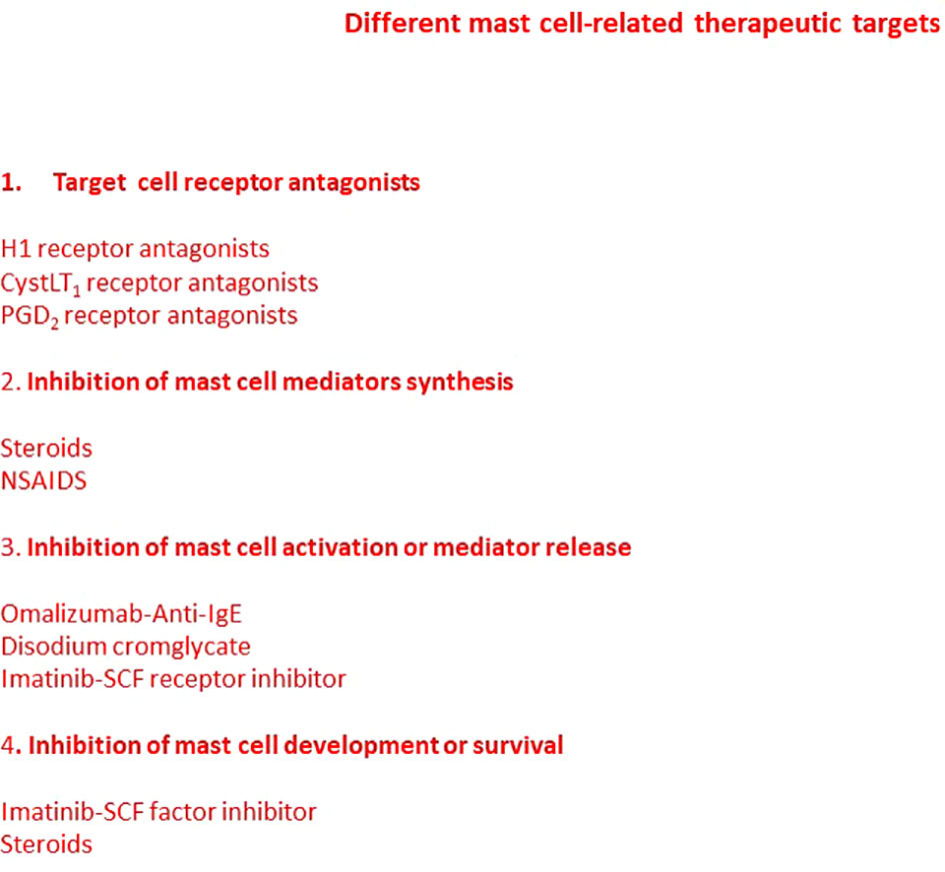
Mast cell-related therapeutic targets. Many pharmacological agents have been developed able to modulate mast cells functions. Some drugs block mediator receptors on target cells, inhibit mast cell mediator synthesis, block mast cell activation or mediator release, or inhibit mast cell development and maturation. Some drugs may act through more than one mechanism. However, new strategies and targets are required.

In conclusion, mast cells may be regarded as a new target for adjuvant treatment adjuvant of CHL through the inhibition of angiogenesis and tissue remodeling and allowing the secretion of cytotoxic cytokines, such as TNF (**Figure 4**).

## Author contributions

DR conceived, planned, and write the work. RT, TA, GI and GS performed the experimental work and revised the ms. All authors contributed to the article and approved the submitted version.

## Funding

This work was supported by Associazione “Il Sorriso di Antonio,” Corato, Italy, and Associazione Italiana Contro le Leucemie, Linfomi e Mielomi (AIL), Bari, Italy.

## Conflict of interest

The authors declare that the research was conducted in the absence of any commercial or financial relationships that could be construed as a potential conflict of interest.

## Publisher’s note

All claims expressed in this article are solely those of the authors and do not necessarily represent those of their affiliated organizations, or those of the publisher, the editors and the reviewers. Any product that may be evaluated in this article, or claim that may be made by its manufacturer, is not guaranteed or endorsed by the publisher.
